# Review of Improvement in Enhanced Access Services for Mental Health Emergencies in NHS Grampian

**DOI:** 10.1192/bjo.2024.406

**Published:** 2024-08-01

**Authors:** Jumoke Ojo, Dharmesh Rai

**Affiliations:** 1 NHS Grampian, Aberdeen, United Kingdom; 2 NHS Grampian, Aberdeen, United Kingdom

## Abstract

**Aims:**

1. Identify changes in the services rendered in the Enhanced access/emergency service following the previously suggested modifications.
2. Identify areas of possible improvement within the service to provide seamless emergency and out-of-hours mental health support to patients.
3. Evaluate adherence to current guidelines for the Enhanced access/emergency service.

**Methods:**

An audit of a total of 100 patients on the list was selected in chronological order. Patient documentation was reviewed against the current criteria for patients on the list, which included having a documented care plan in place, remaining open to a community mental health team, and having been reviewed at least within the last 6 months.

The data was then analysed and compared with the previous year's results to see if there was any significant change year over year.

**Results:**

Year on Year improvement:

1. Total number of patients on the list had increased by 16.7%.
2. The number of patients without a care plan on the list reduced by 6.
3. The number of discharged patients on the list was also reduced by 1.
4. The number of patients who had not been reviewed in six months reduced by 9.

**Conclusion:**

While there had been some improvement in the service provision and adherence to the guidelines, there was still ample room for improvement, which would be achieved by adherence to the guidelines and protocols, to ensure better service improvement for enhanced access and out-of-hours emergency services to patients.

